# Dual drug delivery platforms for bone tissue engineering

**DOI:** 10.3389/fbioe.2022.969843

**Published:** 2022-09-12

**Authors:** Anupama Devi V. K., Sarbajit Ray, Udita Arora, Sunrito Mitra, Alina Sionkowska, Amit Kumar Jaiswal

**Affiliations:** ^1^ Tissue Engineering Group, Centre for Biomaterials, Cellular and Molecular Theranostics (CBCMT), Vellore Institute of Technology (VIT), Vellore, India; ^2^ School of Bio Sciences and Technology (SBST), Vellore Institute of Technology (VIT), Vellore, India; ^3^ Nicolaus Copernicus University, Torun, Poland

**Keywords:** bone tissue engineering, dual drug delivery platform, growth factors, bone regeneration, scaffolds, osteogenesis

## Abstract

The dual delivery platforms used in bone tissue engineering provide supplementary bioactive compounds that include distinct medicines and growth factors thereby aiding enhanced bone regeneration. The delivery of these compounds can be adjusted for a short or prolonged time based on the requirement by altering various parameters of the carrier platform. The platforms thus used are fabricated to mimic the niche of the bone microenvironment, either in the form of porous 3D structures, microspheres, or films. Thus, this review article focuses on the concept of dual drug delivery platform and its importance, classification of various platforms for dual drug delivery specific to bone tissue engineering, and finally highlights the foresight into the future direction of these techniques for better clinical applications.

## 1 Introduction

Bone tissue engineering is an emerging field, in which various physical, chemical, and biochemical signals such as growth factors, drugs, genetically synthesized materials, and small molecules are delivered via a biomaterial-based platform or scaffold to enhance bone regeneration in the affected region (Mottaghitalab et al., 2015; Samorezov and Alsberg, 2015). Regeneration of bone tissues relies on two main processes: osteogenesis and angiogenesis. The former is the assembly of osteoblast cells for extracellular matrix (ECM) deposition and the latter is the vascularization of the bone tissue. These processes are hinged on the activity of the respective growth factors like platelet-derived growth factor (PDGF), fibroblast growth factors (FGF), transforming growth factor–beta (TGF-β), insulin-like growth factor (IGF), bone morphogenetic proteins-2 (BMP-2) and bone morphogenetic proteins-7 (BMP-7), which implies that the delivery of growth factors is vital for tissue regeneration (Samorezov and Alsberg, 2015). This is accomplished by incorporating it into a variety of scaffolds, which attempt to mimic the niche of the bone tissue to enhance regeneration.

The scaffolds, one among the triad of tissue engineering can be fabricated with different biomaterials such as biopolymers like collagen, chitosan, alginates, bio ceramics like hydroxyapatite, nanoparticle-based systems like nanofiber and nano capsules, microspheres and microcapsules, and also gel and film-based materials like gelatin, hydrogel, and poly electrolyte films, etc. These materials are used as they enhance the properties of the scaffold in terms of affinity, structural integrity, and efficiency towards bone formation and growth. These platforms have been experimented with and studied, to infer therapeutic potential and also characterized to get in-depth knowledge of their properties (Grigore and Me, 2018). In many cases, the scaffold itself couldn’t achieve the complete regeneration of bone, which lead to the approach of supplementary delivery of growth factors and drugs that accelerate the process of regeneration thereby the healing process.

**FIGURE 1:**
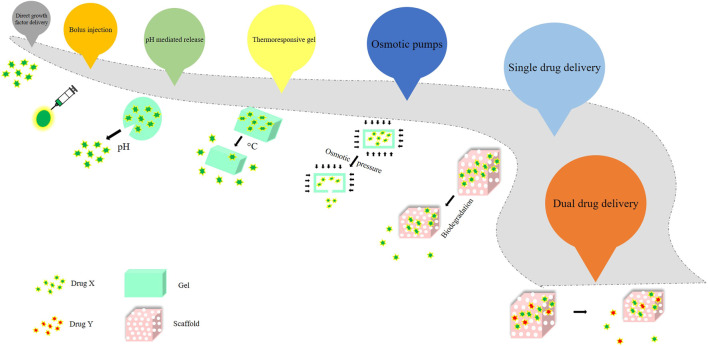
Evolution of drug delivery over years.

The direct growth factor delivery, which was promising fell short of getting commercialized. However, BMP-2 and BMP-7 were approved by the Food and Drug Administration (FDA) to be used instead of autografts (Nauth et al., 2011). Subsequently, other delivery methods and platforms came into existence, such as bolus injection, pH - mediated release, the release of surface-adsorbed protein, thermoresponsive gel, osmotic pumps, and sequential and controlled release by scaffold biodegradation. To obtain therapeutic success in a certain case, it was imperative that the release of the drug was expected to be sustained or prolonged, sequential or biphasic according to need, and the scaffold should mimic a near - natural environment for higher efficacy (Chen and Mooney, 2003; Rambhia and Ma, 2015). The evolution of drug delivery over the years has been represented in Figure 1.

Single drug delivery was explored to regenerate bone tissue, but it was not promising, which led to the emergence of control release of a single drug, which was not efficient because bone tissue is a complex system and its formation and regeneration involve the regulated release of multiple bioactive molecules. Thus, to mimic the niche or the natural environment, scientists turned towards dual drug delivery platforms for bone tissue engineering (Kolambkar et al., 2011). With the alarming rise in bone-related disorders, the most dangerous of which is osteoporosis, a viable therapeutic application of bone tissue engineering is urgently needed. More study into the dual drug delivery platform in bone tissue engineering, which holds the key to promising therapeutic applications in bone formation, has opened up as a result of this. The scaffold manufacturing and design, which includes the growth factors and plays a vital role in their release, is the most crucial aspect of this system. It also helps with quicker regeneration by simulating the microenvironment.

This review focuses on classifying various platforms for dual drug delivery specific to bone tissue engineering applications. The platforms have been classified into five major categories, with descriptions of the fabrication of various scaffolds as well as elucidating the results of dual drug delivery in terms of bone regeneration. Finally, with a critical review of the categories of delivery platforms, the downfalls and the challenges faced are been discussed.

## 2 Bone healing mechanism and tissue engineering approaches

### 2.1 Mechanism of bone repair

Intramembranous and endochondral ossification are the two types of ossification (bone formation) that occur. The adherence of mesenchymal progenitor cells that differentiate into osteoblasts and further mature into bone, such as the mandible and clavicle, is an example of intramembranous ossification. In the latter, mesenchymal progenitor cells attach and grow into chondrocytes, which create cartilaginous templates that are eventually mineralized and replaced by bone. The endochondral pathway, however, is the path by which the majority of our bones are formed (Frohlich et al., 2008).

**FIGURE 2:**
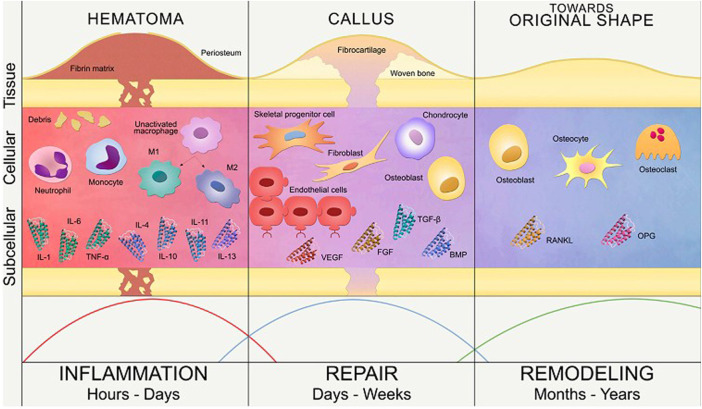
Phases of bone healing in the subcellular, cellular and tissue level (Reproduced with permission from Ref (Lafuente-Gracia et al., 2021) under Creative Commons Attribution License (CC BY)).

In the case of a fractured bone, the first step of bone healing after bone fracture and blood vessel damage is the hematoma formation in the inflammatory phase (tissue level) which serves as a fibrin network that gives the signal for the adhesion and proliferation of angiogenic and chondrogenic progenitor cells. The healing is initiated by the combined effect of various soluble growth factors like ILs, TNF-α, FGFs, BMPs, PDGF, VEGF (subcellular level), and attachment of various cells such as progenitor cells, fibroblast cells, endothelial cells, osteoclast cells and chondrocyte cells (cellular level) to the fibrin network as depicted in Figure 2 (Mottaghitalab et al., 2015).

A brief description of various phases involved in the process of healing a fractured bone is as follows:

#### 2.1.1 Inflammatory phase

It occurs soon after the injury marking the initiation of the healing process, where the hematoma is formed at the site of injury and fills the fracture area thus acting as a scaffold for cellular mobility (Ghiasi et al., 2017). The inflammatory cells such as macrophages, monocytes, lymphocytes, and fibroblasts infiltration cause granular tissue formation that results from the ingrowth of vascular tissue (Kalfas, 2001). Several inflammatory mediators, including interleukins (IL-1, IL-6, IL-11, IL-18), and TNF are considerably released during this phase, or a few days following the injury. The macrophages at the site of fracture secrete various growth factors that accelerate bone healing by aiding the process of cell migration and proliferation. These include growth factors such as BMP-2, BMP-5, BMP-7, FGF, IGF that aid in various functions such as cell recruitment, migration, and proliferation of MSCs (Oryan, Monazzah and Bigham-Sadegh, 2015).

#### 2.1.2 Reparative phase

The fibroblasts then begin to lay stroma cells which helps in vascular ingrowth (Einhorn and Gerstenfeld, 2015). With the progress in vascular ingrowth, the collagen is laid followed by the secretion of osteoid and their mineralization (Kalfas, 2001). The formation of soft callus, the onset of angiogenesis, and the formation of connective tissue define this phase, which is followed by the replacement of soft callus by woven bone in the following stages (Oryan, Monazzah and Bigham-Sadegh, 2015). The progenitor cells originate from bone marrow, and travel to the damaged region after receiving the signal from hematoma; leading to the formation of soft callus. Therefore, by developing a callus that later goes through chondrogenesis analogous to an endochondral pathway, external soft tissue aids in stabilising the fracture. Soft callus becomes hard callus after the remodeling of proliferative cartilage into hypertrophic cartilage, revascularization, matrix mineralization, and woven bone production. These functions are been aided by TGF- β, BMP-2, BMP-5, BMP-6, PDGF, and IGF (Oryan, Monazzah and Bigham-Sadegh, 2015).

#### 2.1.3 Remodeling phase

**FIGURE 3:**
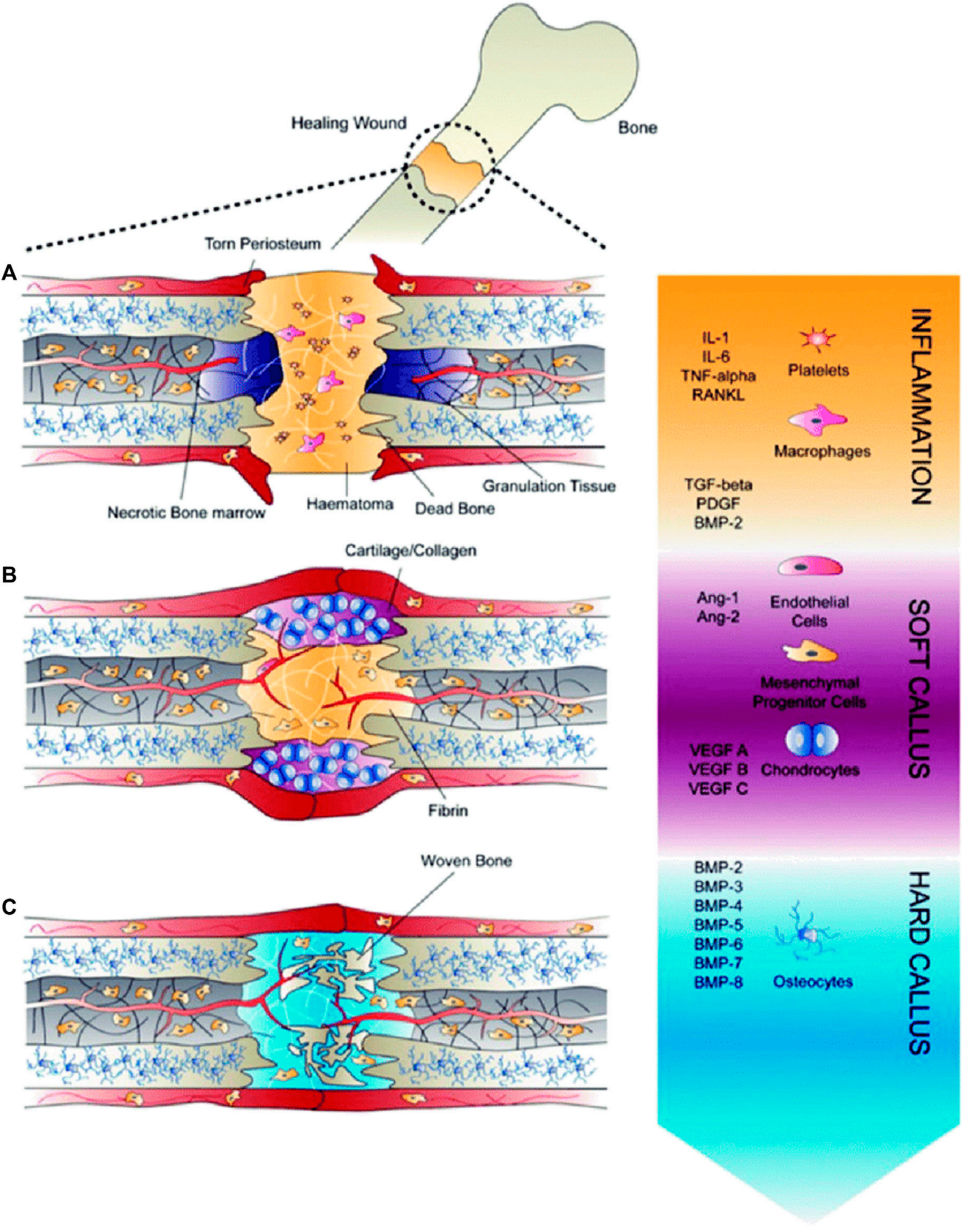
Healing of fractures **(A)** Inflammatory phase, **(B)** Soft callus development, during which angiogenesis occurs, and **(C)** Hard callus formation, during which growth factors induce differentiationof recruited mesenchymal progenitor cells (Reproduced with permission from Ref (De Witte et al., 2018) under Creative Commons Attribution License (CC BY)).

This phase entails the mineralization of the callus and its replacement with mineralized bone, restoring the callus’ original shape, size, and mechanical qualities. The woven bone is resorbed by osteoclasts, and the matrix is replaced by osteoblasts with lamellar bone (Oryan, Monazzah and Bigham-Sadegh, 2015). The remodeling phase is successful when there is a balance between woven bone resorption and lamellar bone deposition, as well as with an appropriate supply of blood and mechanical stability. Although the process is initiated in the third to fourth week, it takes from months to years for the process to remodel a complete bone structure (Marsell and Einhorn, 2011). Remodeling of bone gives mechanical properties to the bone which reacts to different loading or stress conditions (Amini, Laurencin and Nukavarapu, 2012; Schell et al., 2017). During this phase, there are elevated levels of TNF, interleukins, and is also aided by growth hormone and parathyroid hormone enhancing the healing and strengthening of fractured callus (Oryan, Monazzah and Bigham-Sadegh, 2015). The entire process of bone healing has been represented in Figure 3. We can therefore infer from this concise explanation of the mechanism of bone healing that different growth factors and molecules are involved throughout different stages of healing, therefore substantiating the need for multiple growth factors from drug delivery systems.

### 2.2 ECM of bone tissue

A three-dimensional, non-cellular material called an ECM is what provides a tissue its flexibility and integrity. Osteoblasts synthesize it before bone mineralization. The composition of the ECM varies from tissue to tissue and is influenced by a variety of factors including age, illness, and cellular diversity. ECM deposition aids cell differentiation and proliferation even more. It consists of 40% organic and 60% inorganic components. Organic ECM is more complicated, with collagen (90%) and non-collagenous protein (the remaining 10%). Collagen types I, III, and V are the primary components of collagenous protein (Carvalho et al., 2018; Schlesinger et al., 2020; Carvalho and Cabral, 2021). Collagen fibrils inter and intra crosslinks supply the ECM with the mechanical support it requires, acting as a scaffold.

**FIGURE 4:**
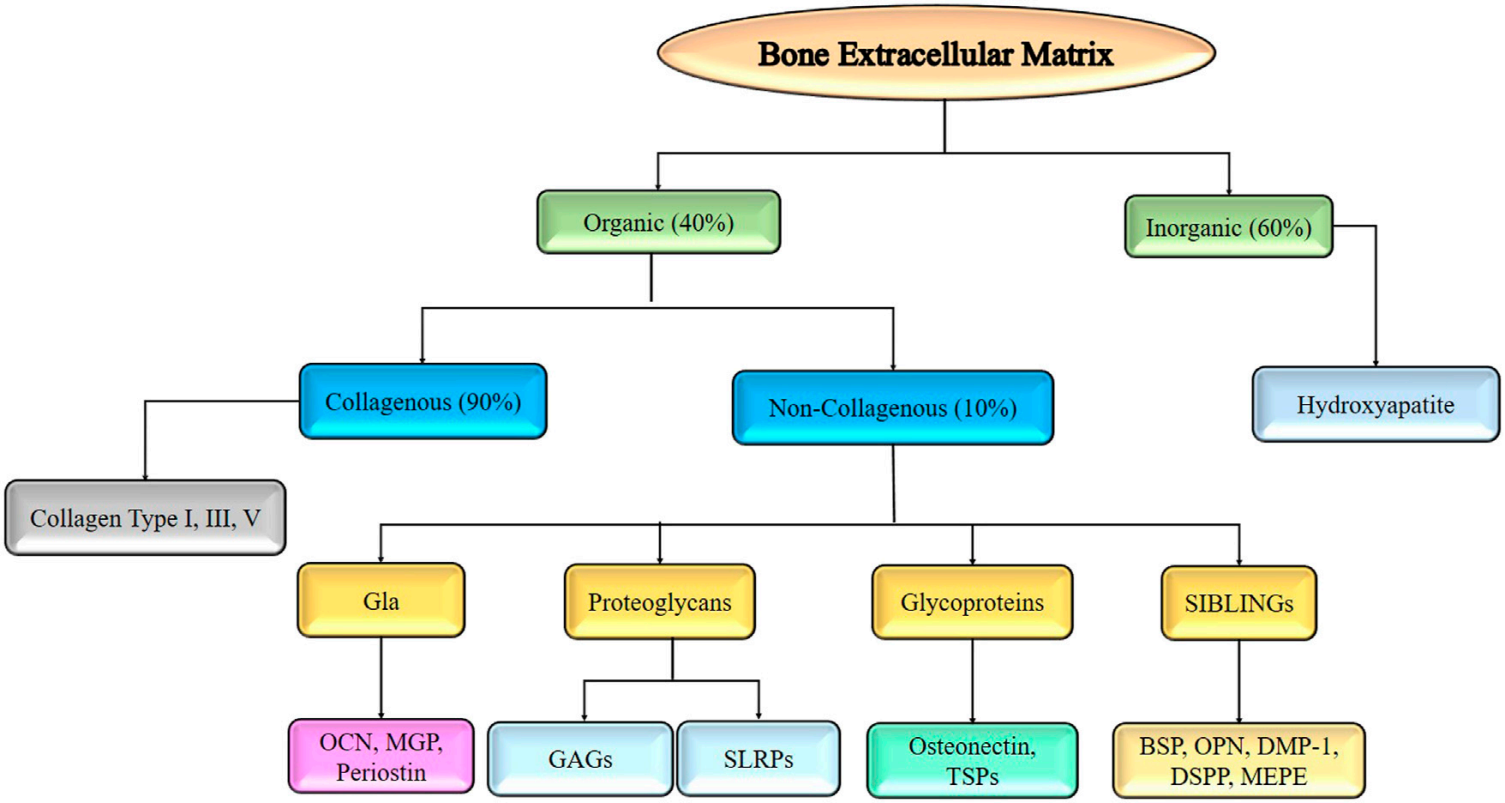
Components of bone extracellular matrix required for developing tissue engineered scaffold for bone regeneration.

Proteins containing y-carboxyglutamate, proteoglycans, glycoproteins, and tiny integrin-binding ligands are the four subcategories of non-collagenous proteins. The glycosaminoglycans (GAG) keratan sulfate, chondroitin sulfate, heparan sulfate, hyaluronic acid, and dermatan sulfate are examples of glycosaminoglycans, whereas osteocalcin (OCN), matrix Gla protein (MGP), and periostin are ECM protein components that contain carboxyglutamic acid (Gla). (Carvalho and Cabral, 2021). Small leucine-rich proteoglycans (SLRPs), which include biglycan, decorin, keratocan, and aspirin, are proteoglycan families found in the ECM of bones. The presence of osteonectin and thrombospondins (TSPs) is usually indicative of glycoprotein. Bone sialoprotein (BSP), osteopontin (OPN), dentin matrix protein-1 (DMP1), dentin sialophosphoprotein (DSPP), and matrix extracellular phosphoglycoprotein (MEPG) are all members of the SIBLING family of glycophosphoproteins (MEPE). The inorganic constituents of bone mainly include hydroxyapatite (HA, Ca_5_(PO_4_)_3_OH) as the major constituent (Kolb and Bussard, 2019; Lin et al., 2020a). These constituents of the bone extracellular matrix are represented in Figure 4.

Growth factors are also present in the microenvironment of tissues, which help in bone regeneration. They are either autocrine (molecules act on cells that produce them) or paracrine (molecules act on nearby cells). These growth factors help in signaling by binding to the surface receptors of producing cells or nearby cells, which thereby propagates the signal with the help of secondary messengers to the interior of the cells and regulates cell proliferation, differentiation, and enhances ECM formation. There are various types of cells and growth factors that contribute differently to tissue healing and regeneration. TGF links with the ECM to control how MSCs differentiate into osteoblast cells and osteocytes, which continue to differentiate with the secretions of the ECM. The cytoskeleton’s organization, osteoclast cells’ behavior, cellular morphology, and fibronectin’s morphology are all significantly influenced by the stiffness of the ECM (Lin et al., 2020b).

A detailed description of the release of drugs (growth factors, drugs, chemoattractant, genes) by tissue engineering techniques has been discussed in the fore coming sections.

### 2.3 Factors involved in osteogenesis and angiogenesis

Osteogenesis is the process in which the progenitor cells transform into osteoblast cells, which deposit ECM components such as glycoproteins and proteoglycans with GAGs. Growth factor adhesion and morphogenetic function are assisted by GAG molecules and other ECM components. The most common osteogenic factors identified are PDGF, TGF-β, FGF, IGF, and BMPs. Out of all these, BMP-2 is considered the most widely used growth factor for signaling and expressing osteogenic markers. These factors have aided tissue engineering approaches and resulted in effective neo bone formation (De Witte et al., 2018).

The formation of new blood vessels from preexisting vascular systems is known as angiogenesis. It is one of the necessary phenomena in bone regeneration. Blood vessels have an important role not only in transporting oxygen and nutrients but also in acting as a conduit for additional osteoblasts. This helps with cell differentiation and endochondral ossification. The formation and proliferation of blood vessels are triggered by endothelial cell migration across the vascular system. PDGF, BMPs, FGFs, and TGF-β are some of the most important pro-angiogenic factors identified (Kim and Tabata, 2015; De Witte et al., 2018). Studies were carried out with the delivery of one or more of these growth factors to access the impact of it in improving the rate of bone healing. Investigations into accompanying one or more of the growth factors are still ongoing, to mimic the environment and release these factors at the site of injury to aid in bone regeneration.

The summary of the growth factors that aid osteogenesis and angiogenesis are discussed with their brief functions in Table 1. A brief description of growth factors and their role in bone healing and formation are discussed as follows:

**TABLE 1:** Summary of various growth factors and their functions that promote osteogenesis and angiogenesis.

Process	GFs	Functions	References
Osteogenesis	PDGF	Primary signal for cell attachment to the fracture area, enhances bone formation	(Andrades et al., 2013
			De Witte et al. (2018)
	TGF-β	Inhibit osteoclast formation and bone resorption, Activates fibroblasts and preosteoblasts	Andrades et al. (2013)
	FGF	Mature chondrocytes and osteoblasts, Maintains balance between osteoblasts and osteoclasts	Andrades et al. (2013)
	IGF	It serves as a mitogenic factor, promoting the growth and proliferation of osteoblasts	De Witte et al. (2018)
	BMP-2	Boost the number of osteogenic progenitor cells and their differentiation	(Zhang et al., 2014
			De Witte et al. (2018)
	BMP-7	Leads to chondrogenesis and osteogenesis of adipose derived stem cells	Zhang et al. (2014)
	SDF-I	Assist In the early stages of fracture for stem cells recruitment and migration	De Witte et al. (2018)
Angiogenesis	VEGF	Stimulates migration and proliferation of endothelial cells and hence promotes neovascularization	(Geiger et al., 2005
			Andrades et al. (2013)
	PDGF	Recruits MSCs, promotes chemotaxis and angiogenesis, Induces mitosis of endothelial cells	(Andrades et al., 2013
			De Witte et al. (2018)
	BMP	Increasing endothelial motility and invasion by promoting proliferation	Pi et al. (2012)
	FGF	Proliferation of endothelial and osteoblasts cells	Andrades et al. (2013)
	TGF-β	Enhance proliferation and differentiation of MSCs, activates fibroblast to lay down collagen	Andrades et al. (2013)

#### 2.3.1 TGF- β

Transforming growth factor- β, a multifunctional growth factor released by the pericardium and periosteum and widely found in platelets, with bone being the most abundant source (200 μg/kg tissue), is essential for bone remodeling (Kempen et al., 2010). This is a 25-kDa protein that increases matrix protein synthesis in bone cells, which affects bone growth and resorption. Depending on the phenotypic and/or development stage of bone cells, it has a variety of consequences. This growth factor has a direct effect on osteoblasts (chemoattractant), encouraging differentiation or proliferation during endochondral ossification (Makhdom and Hamdy, 2013). It suppresses the production of osteoclast precursors and bone resorption and has inhibitory effects on isolated osteoclasts, the cells that cause bone resorption, at higher concentrations. It functions as a bone-coupling factor, bridging the gap between bone resorption and deposition (Andrades et al., 2013). It also promotes collagen formation and upregulates the expression of non-collagenous ECM proteins involved in bone turnover and mineralization regulation (Makhdom and Hamdy, 2013).

#### 2.3.2 PDGF

**FIGURE 5:**
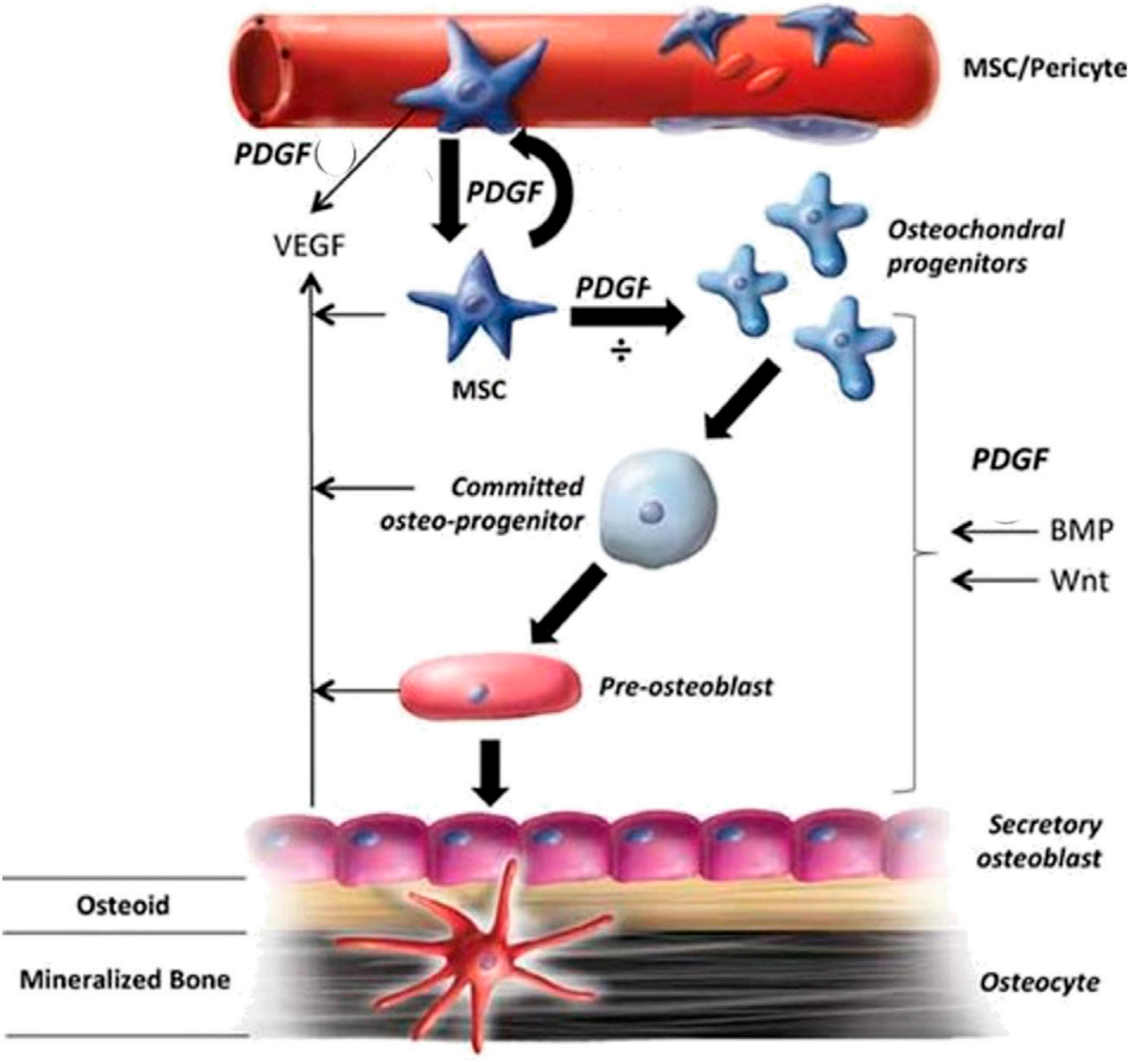
The role of PDGF in bone healing and the mechanisms involved (Reproduced with permission from Ref (Caplan and Correa, 2011). Copyright ^©^ 2011 Orthopaedic Research Society).

The two-chain polypeptide PDGF is produced by macrophages and stored in platelets exists in various isoforms such as PDGF-AA, PDGF-AB, PDGF-BB, PDGF-CC, and PDGF-DD. PDGF-BB is a potent mitogen and chemoattractant that interacts with all three PDGF receptors: PDGFR-αα, PDGFR-αβ, and PDGFR-ββ (Zhang et al., 2018). It is considered to be a key regulator in tissue repair and reconstruction, regulating fracture healing and promoting angiogenesis (Sun et al., 2021). PDGF is released as platelets aggregate at the fracture site; it then diffuses into the environment and functions as a chemoattractant, thereby recruiting cells. This raises the number of stem cells, which are then activated and transformed into osteoblast cells. This results in upgraded bone growth (Figure 5) and is one of the major contributing factors to cellular infiltration into the fracture site. Its efficacy is limited due to its short life duration of only a few minutes (Shah N et al., 2014).

#### 2.3.3 FGF

The fibroblast growth factor (FGF) family of 22 different members are monomeric proteins of 16–18 kDa. They have various biological functions that include roles in mitogenesis, cellular migration, differentiation, angiogenesis, wound healing, and bone development (Yun et al., 2012; Charoenlarp, Rajendran and Iseki, 2017). Along with promoting angiogenesis, it has the ability to promote osteocyte proliferation and is a potent MSC mitogen. It also has a short life duration of 90 s, finding it challenging to deliver, necessitating the use of a delivery platform for long-term distribution (De Witte et al., 2018).

#### 2.3.4 IGF

Growth hormones released by the pituitary glands cause target cells in the liver to secrete IGF, which is a single-chain polypeptide with a molecular weight of 7.5 kDa (Kempen et al., 2010). Estrogen, parathyroid hormone, cortisol (which suppresses IGF-I production), local GFs, and cytokines all influence its secretion. IGF-I and IGF-II are the two types of growth factors found in the bone matrix, with IGF-II being the most abundant. IGF-1 is a mitogenic agent that promotes embryonic cell development and differentiation, which in turn promotes osteoblast growth and proliferation (Andrades et al., 2013). During the endochondral bone formation phase, IGF- II promotes the synthesis of cartilage matrix, type I collagen synthesis, and elevate cellular proliferation. IGF is necessary for bone formation, healing, and the proliferation and differentiation of MSCs (Kempen et al., 2010; Yun et al., 2012; Makhdom and Hamdy, 2013).

#### 2.3.5 BMPs

These proteins are structurally related to TGF- β. They promote osteoprogenitor cell proliferation and differentiation, resulting in bone formation. The only BMPs that are commercially accessible and have received clinical approval are BMP-2 and BMP-7. (Nauth et al., 2011). It is a component of FDA-approved bone regeneration systems and has been used in the clinic to treat open tibia fractures, non-union bone injuries, and spinal fusion. It is a key player in the bone healing cascade, principally via stimulating osteogenic differentiation of osteoprogenitor cells and the recruitment of MSCs to the fracture site (De Witte et al., 2018). It has the potential to stimulate the growth of new bone and repair bony defects (Chen and Mooney, 2003).

#### 2.3.6 SDF

The well-known chemokine stromal cell-derived factor is involved in the recruitment of circulating hematopoietic and mesenchymal stem cells, which contributes to overall vascularization and bone regeneration (Kim and Tabata, 2015). It is also induced in the periosteum of the injured bone thereby recruiting MSCs to the fracture site thus promoting endochondral ossification (Liu et al., 2013). Through several studies, it has been found that this growth factor is associated with angiogenesis and thus directly impacts osteogenesis (Yang et al., 2018). Studies by Fang Yang et al., have proved that overexpression of SDF assists in the regeneration of bone by inducing angiogenesis and further aid in osteogenesis (Yang et al., 2018). It has also been used in studies involving a segmental bone defect in the radial bone of rabbits by Guobao Chen et al., and has been concluded that the inclusion of SDF enhanced osteogenesis (Chen and Lv, 2017).

#### 2.3.7 VEGF

The 34–46 kDa glycoprotein, the vascular endothelial growth factor is expressed within the first 2 weeks after a bone fracture (Kanczler and Oreffo, 2008). These are heparin-binding growth factors that are expressed by osteoprogenitor cells and chondrocytes, allowing bone cells such as osteoblasts and osteoclasts to be recruited alongside hematopoietic cells. It is crucial at the fracture site throughout the healing process as it promotes osteoblast differentiation and facilitates endothelial cell migration and proliferation. As these bone cells are attracted to the site of injury, this is the first stage of the bone formation process. They also play a vital role in inducing angiogenesis (Geiger et al., 2005). Excess VEGF secretion, on the other hand, would result in bone resorption due to an increase in osteoclast recruitment (Diomede et al., 2020).

### 2.4 Tissue engineering approaches

**FIGURE 6:**
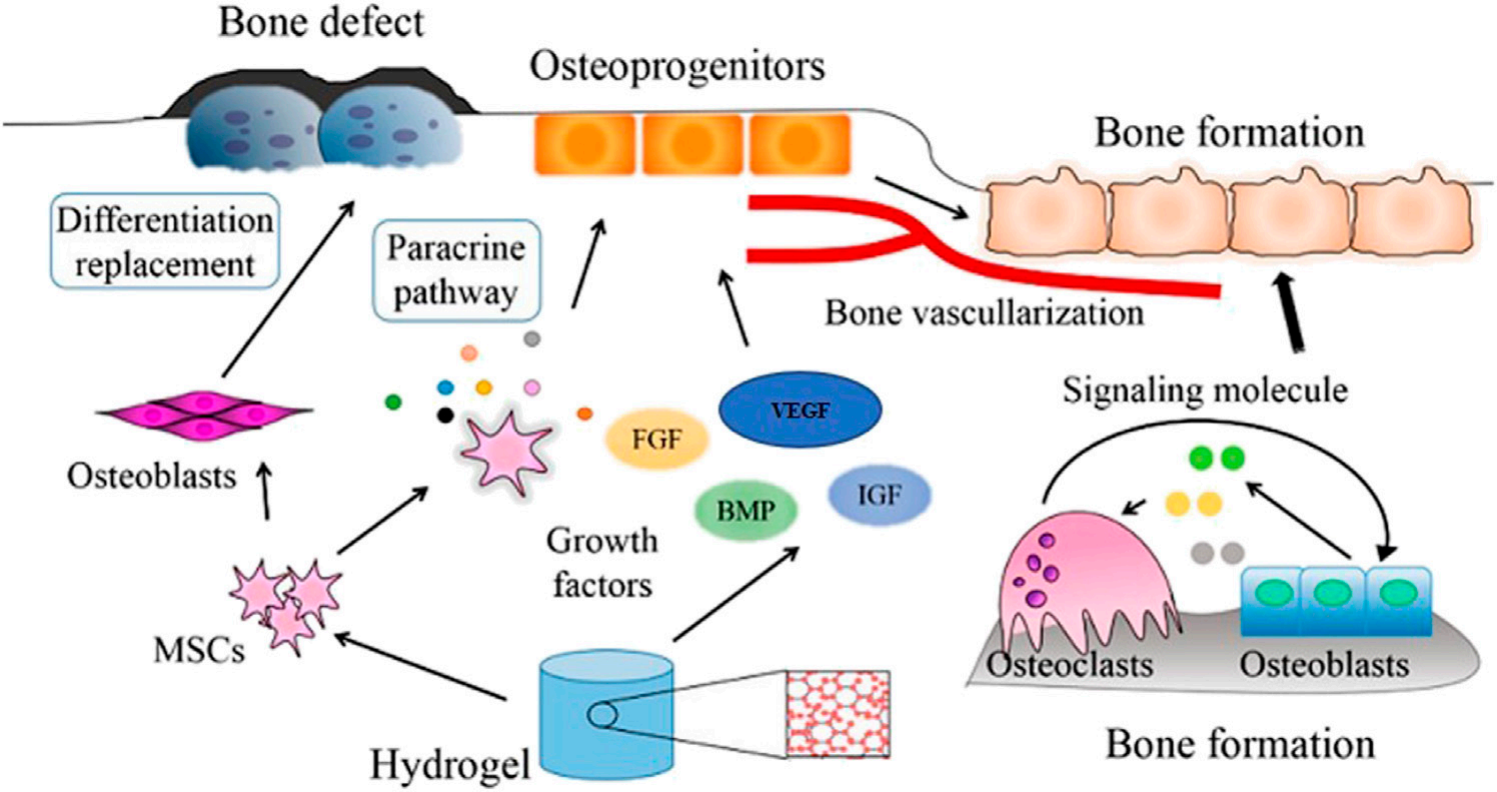
Mechanism of action of cells and growth factors incorporated into a bone-repair scaffold (Reproduced with permission from Ref (Yue et al., 2020) under Creative Commons Attribution License (CC BY)).

Bone is crucial in ensuring and supporting posture, mobility, and locomotion. It is also necessary for maintaining other physiological features such as blood cell production, homeostasis, pH maintenance, and mineral reserve (Porter, Ruckh and Popat, 2009). Consequently, damaged bone tissue influences many functions in our body, persuading scientists to conduct more research and development in bone tissue engineering, a multidisciplinary branch of study aimed at producing osteogenic bone implants for tissue regeneration (Fernandez-Yague et al., 2015). Reports elucidate that around billions of dollars are spent on bone grafting on millions of people annually. For example, a report from Europe and the United States shows that more than 4,00,000 and 6,00,000 persons, respectively, have been affected and seek bone grafts each year. In reference to the World Health Organization (WHO), the cost of caring for people with musculoskeletal defects in the United States in 1995 was $215 billion, which is expected to be exponential in the fore coming years (Mottaghitalab et al., 2015). Over 2 million bone transplant procedures are estimated to be performed each year, costing over $2.5 billion globally (Samorezov and Alsberg, 2015). But the technique remains arduous, and the complexities of the bone tissue ultimately reduce the chances of success. Multiple surgeries, donor scarcity and morbidity, and high failure rates are the other possible constraints to autograft implementation. Although autografting is the most common treatment for damaged bone tissue, active research on various therapeutic approaches or treatments is in progress (Kim et al., 2009). One such is the advancements in the fabrication of biomaterial-based scaffolds that are used widely to replace the limitation of non-availability of autografts. All scaffold-based tissue engineering can’t induce growth of the tissues. To overcome this constraint, the idea of employing these scaffolds as a platform for delivering molecules or growth factors that aid in tissue regeneration arose, intending to stimulate the tissue’s microenvironment (Figure 6).

The natural mechanism of bone repair is emulated by tissue engineering techniques in case of defect size. This technique employs scaffolds, designed by mimicking the ECM to achieve the microenvironment for enhanced bone tissue regeneration. The dual drug delivery system evolved as a more significant technique as compared to the single drug delivery system since there are a few molecules or growth factors that play a key part in this process.

## 3 Dual drug delivery and its importance

There are several techniques adopted for the controlled release of the drugs, growth factors, and other low molecular weight molecules from the scaffold. One of the techniques that have shown its enumerated benefits is dual drug delivery. It has aided the process of bone tissue regeneration by allowing two growth factors (drugs) to be delivered concurrently and in a regulated manner from the scaffold (Kim and Tabata, 2015). It tries to mimic the microenvironment of tissue where cells release multiple growth factors at a time to heal the tissue or regenerate it.

To rebuild bone tissue, two methods could be used: cell implantation and tissue engineering. Cell implantation can be an effective method for normal fracture healing or some small-sized defects (Dimitriou et al., 2011). As the growth factors or cells have been injected into the degradation site, they are likely to get digested or leave the site earlier without their action showing its short life span (Rambhia and Ma, 2015). For example, PDGF, FGF-2, and VEGF have half-lives of 2, 3, and 50 min respectively (Chen and Mooney, 2003). To overcome this possibility, the most suitable therapeutic approach is tissue engineering, where cells or growth factors are incorporated into a scaffold, which results in a controlled release based on the stimuli and has high osteogenic activity. This results in the efficient regeneration of bone tissue. This method can be used to repair major bone defects brought on by trauma, infection, tumour excision, skeletal abnormalities, and situations where the regenerative process is hampered, such as osteoporosis, avascular necrosis, and atrophic non-unions (Dimitriou et al., 2011; Kim and Tabata, 2015; Grigore and Me, 2018).

### 3.1 Dual growth factors delivery system

Growth factors are soluble factors that act on multiple sites in tissue. They are naturally present in the tissue microenvironment, which helps with bone regeneration. When tissue damage and bone defects occur, growth factors degenerate. They are supplied through artificial means. Some of the most important growth factors in bone regeneration are TGF-β, IGF, PDGF, FGF, BMPs, VEGF, and hepatocyte growth factor (HGF). As growth factors often travel from the site of action to other tissues, they are sent to the target tissue through a dual delivery platform, which ensures their controlled release.

When two growth factors are combined, the process of bone repair is accelerated. BMP-2 and VEGF, which are responsible for osteogenesis and angiogenesis, respectively, are the most commonly employed combinations (Patel et al., 2008). In the study by Simon Young et al., these growth factors were loaded onto gelatin and poly (lactic-co-glycolic acid), which serve as carriers for delayed and rapid release, respectively. The fast secretion of BMP-2 with the slow or fast secretion of VEGF has significantly enhanced the process of bone regeneration. But they have concluded that for long-term effective bone regeneration, the optimum loading dose, the ratio of growth factor required, and the release profile need to be optimized (Patel et al., 2008; Young et al., 2009). Other popular combinations include BMP-4 and VEGF, BMP-7 and PDGF, and VEGF and PDGF. All these combinations show significant effects when used together in comparison to only one growth factor with scaffold or scaffold alone (Shung et al., 2002; Kim and Tabata, 2015).

### 3.2 Drug and growth factor (combination) delivery system

Drugs in combination with growth factors are released in a controlled manner to enhance bone regeneration. Hiroshi Kohara et al., employed BMP-2 with Wnt1 inducible signaling pathway protein embedded into gelatin sponges, which resulted in improved osteoid production by differentiating BMP-2-recruited mesenchymal stem cells, showing that Wnt 1 increases BMP-2’s function (Kohara and Tabata, 2011). Another important combination is BMP-2 with triptolide incorporated into a hydrogel and the inhibition of pro-inflammatory cytokine expression like interleukin (IL)-1β and tumor necrosis factor (TNF)-α as these factors on osteogenic differentiation was studied by the research group of Lacey et al. The results revealed that these interleukins play a great role in bone loss and inflammatory conditions like rheumatoid arthritis (Lacey et al., 2009; Kim and Tabata, 2015). In a research work by Lauren S Sefcik and colleagues, they used Sphingosine 1-Phosphate, which is a bioactive phospholipid autocrine and paracrine signaling molecule that helps in increasing the size of microvessels and its number with impact on the migration and proliferation of these cells. They are released from the scaffold of PLGA (Sefcik et al., 2008).

BMP-2 with Dexamethasone (DEX) is the most extensively utilized combination. DEX is a widely used drug that helps in bone regeneration by promoting the osteo-differentiation ability of cells. One of the research was with BMP-2 loaded onto a silk fibroin (SF)/PLGA scaffold by Jihang Yao et al., where the results show that there was an early burst release of DEX with subsequent sustained release of BMP-2 thereby achieving strong osteogenic potential (Yao et al., 2019). The same combination has been used with PLGA/alginate core-shell microcapsules thereby achieving enhanced stem cell differentiation (Choi et al., 2010).

There are various low-molecular-weight drugs supported in one or another following way. One such is statins that proved their action by enhancing the release of BMP-2 by hindering 3-hydroxy-3-methyl-glutaryl coenzyme A (HMG-CoA) reductase (Ibrahim, Mohamed and Shuid, 2013). Lovastatin, one of all the statins was the first statin approved for use in humans (Tobert, 2003). Despite all these drugs with BMP-2, several others like deferoxamine (Yao et al., 2018), dexamethasone, simvastatin, and alendronate (Lee et al., 2021) are commonly used drugs for dual drug delivery that enhances bone tissue regeneration.

### 3.3 Other biomolecules dual delivery system

Bone regeneration can be enhanced by cell recruitment as cells are responsible for ECM secretion and hence regeneration of tissue. These cells can be stem cells, which on differentiation give the required cell types. To achieve cell attachment, chemoattractants along with growth factors are released through a scaffold. Chemo attractants enhance cell recruitment and retention to the tissue damage, which results in faster bone regeneration. The most widely used combination is SDF-1 and BMP-2 incorporated into gelatin hydrogel. SDF-1 is a chemokine (CXC motif) ligand 12 (CXCL) that recruits bone marrow-derived hematopoietic and mesenchymal stem cells that differentiate to increase vascularization and osteogenesis, thereby increasing bone growth (Kim, Lee and Kim, 2018). Dual controlled-release results in faster recovery of bone. Likewise, the combination of Substance P (SP) and BMP-2 incorporated into heparin-conjugated fibrin gel also results in stem cell recruitment and osteogenesis, which helps in faster bone regeneration (Kaneda, 2012; Noh et al., 2015).

As inflammation is the initial step of bone regeneration, inflammatory cell recruitment has become one of the areas of research. Compared to the release of either macrophage recruiting agent or platelet-rich plasma from gelatin hydrogels alone, the combination of an S1P1 agonist and platelet-rich plasma dramatically boosted the number of macrophages that were attracted and elevated the levels of gene expression of anti-inflammatory cytokines (Kim, Furuya and Tabata, 2014).

### 3.4 Drug-Drug (Combined) delivery system

Combined therapy with drugs has various therapeutic effects as they offer an effective method for treating various disorders and aid in tissue regeneration. This is achieved by the release of different drugs at different time intervals in order to maximize their effects, for which the independent release of the drugs remains a major challenge. To overcome this challenge, Lan Wei et al., had designed a scaffold system consisting of hydrogel and micelle composites incorporated with dual drugs named aspirin and doxorubicin. As expected by them, independent release was achieved as aspirin had a short term release whereas doxorubicin exhibited a prolonged and sustained release which is due to the pH dependency of the system (Wei et al., 2009).

Thus, pH plays a vital role in regulating the release behavior of the drugs. To substantiate this, Wei Xia et al., have used the water soluble gentamicin and the fat soluble naproxen where the predominant release of gentamicin can be achieved in the acidic environment, whereas in the basic environment, swift release of naproxen has been observed (Xia et al., 2008).

The fabrication of scaffolds is essential for controlling the release behavior. The scaffold made of core and shell nanofibers is still promising for dual drug delivery since it allows for the loading of distinct drugs in the core and shell as well as aids in their independent release. In order to achieve regulated release, Davood et al., loaded the model drugs diclofenac sodium salt and gentamicin sulphate into the core and shell of the nanofibers (Kharaghani et al., 2019).

Therefore, it might be concluded that the independent release of the various medications can be achieved by altering the environment, the shape of the carrier, or by controlling the solubility of the drugs.

## 4 The interplay of scaffolds and dual drug delivery in bone tissue engineering

**FIGURE 7:**
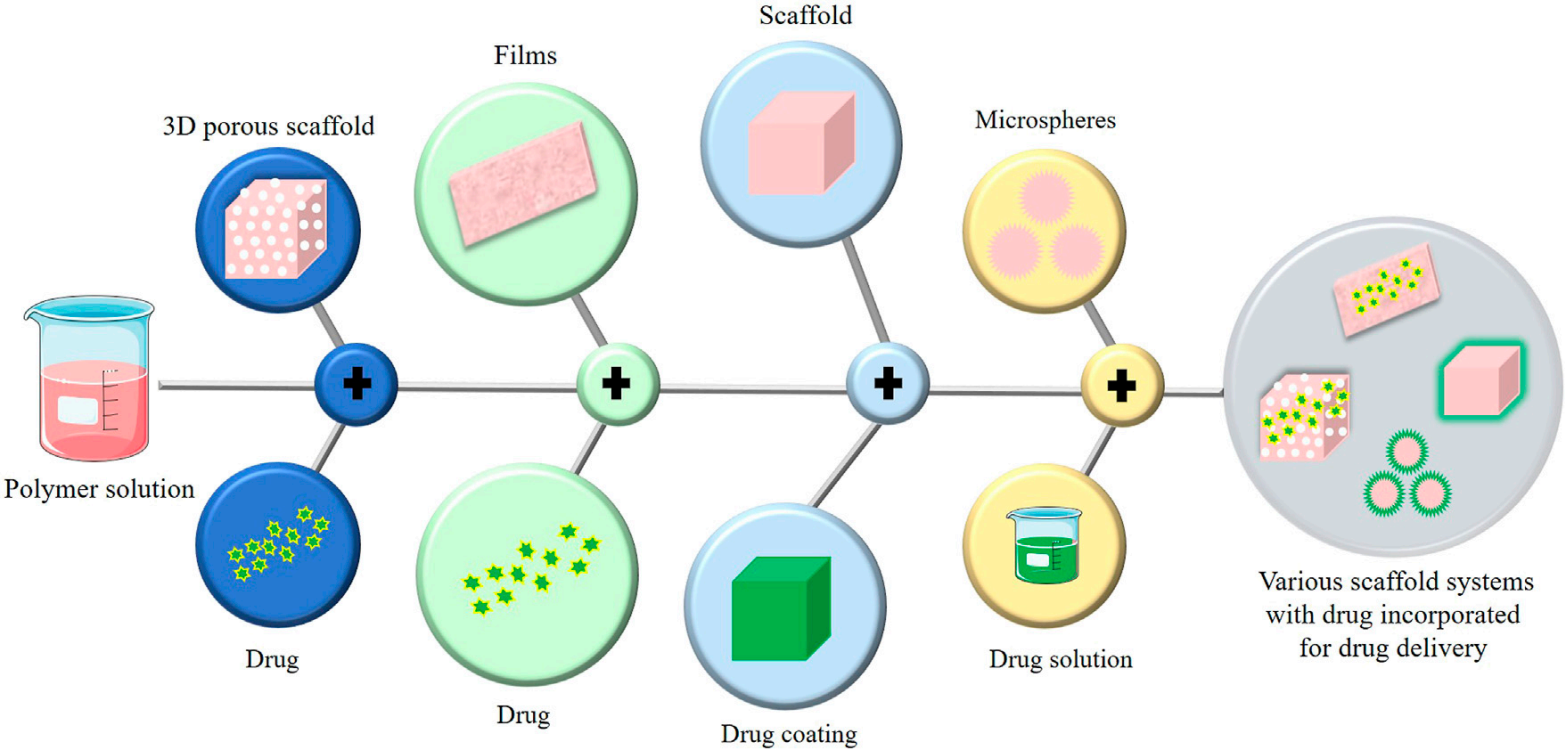
Various scaffold systems for drug delivery.

In conventional tissue engineering, osteogenic cells and bioactive chemicals (growth factors, medications, etc.) are combined into a biomaterial that mimics the milieu for the cells to secrete osteoid, the matrix of newly formed tissue. Scaffolds are designed to imitate the microenvironment of the tissue so that cells can execute the appropriate action. For controlled drug release and optimal bone regeneration, a dual drug delivery system is used. To achieve the same, various types of platforms (Figure 7) have been studied and designed. Some of the categories discussed are 3D porous scaffolds, gel and film-based platforms, scaffolds coated with other materials, microcapsules and microspheres, nanoparticles, nanocomposites, and nano capsules. Each of these platforms are been discussed in the fore coming sessions.

A scaffold exhibiting different biochemical and biomechanical properties, pore size, porosity, and roughness leads to the migration, morphogenesis, differentiation, and homeostasis of the mesenchymal stem cells or osteogenic cells in the required manner. Scaffolding, cells, and growth factors together construct the bone implant (Frohlich et al., 2008).

Biocompatibility is the most important criterion to consider when designing tissue-engineered bone implants. Biocompatibility refers to a biomaterial’s capacity to perform its intended role in a medical therapy while causing no adverse local or systemic effects in the patient. To evaluate the graft’s toxicity and capacity for osteogenesis, osteoconduction, osteoinduction, and osteointegration, several clinical investigations have been conducted (Tzavellas et al., 2020).

## 5 Release modes of drug from scaffolds

**FIGURE 8:**
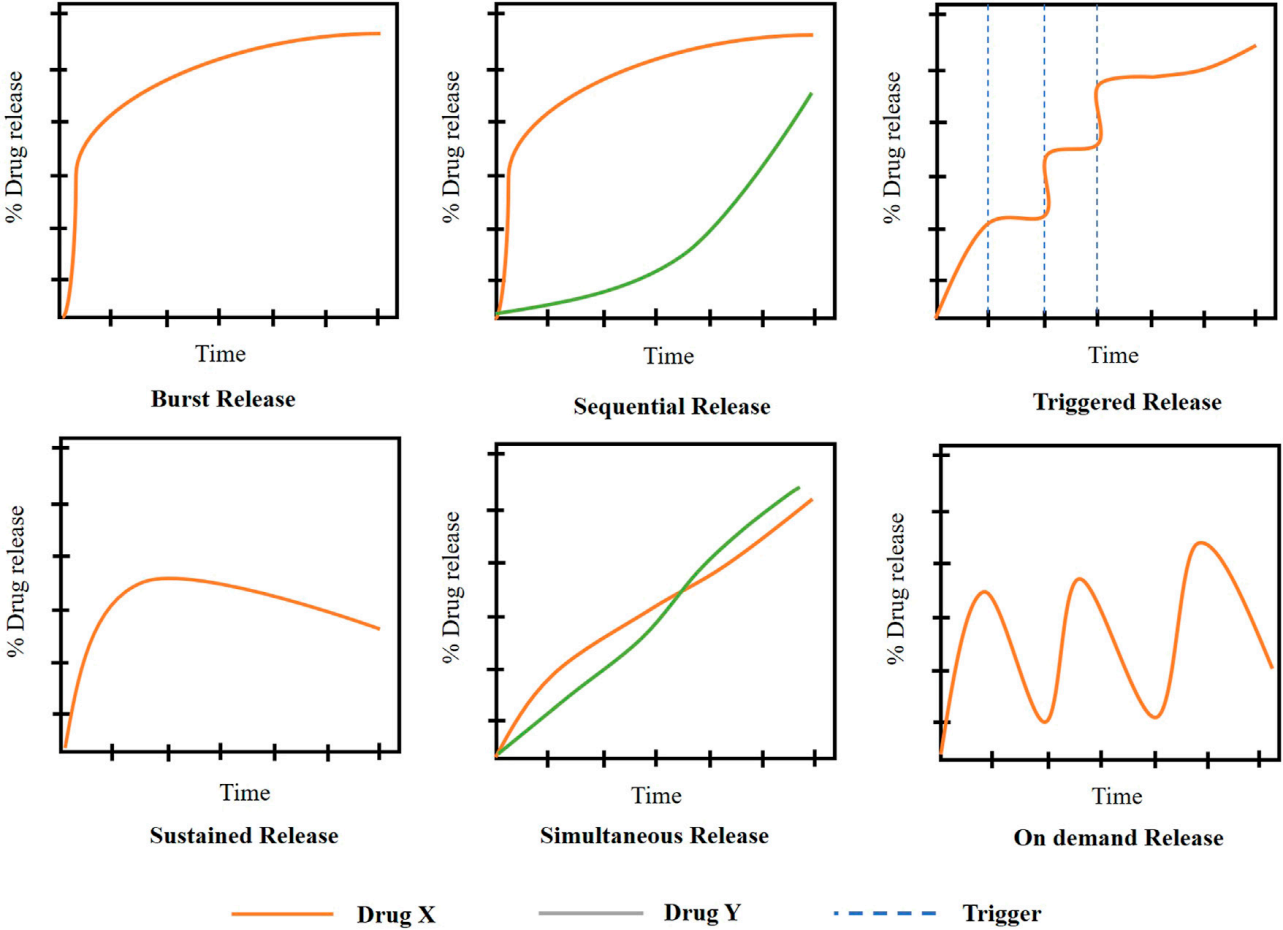
Drug release mechanisms from scaffolds ((Adapted with permission from Ref (Rothe et al., 2020; Adepu and Ramakrishna, 2021) under Creative Commons Attribution License (CC BY)).

The release modes from the scaffold can be altered by the change in the drug loading methodology, the morphology of the scaffold, degree of crosslinking, pore size and porosity and also the degradation rate of the scaffold (Lee et al., 2021). The various modes of drug release (Figure 8) are: Burst release, sustained release, triggered release, simultaneous release, and sequential release.

### 5.1 Burst release

It is an uncontrolled and unforeseen release that causes the sudden delivery of drug initially at higher concentrations followed by the limited drug release further (Bhattacharjee, 2020). In the case of bone tissue regeneration, this mode of release is not preferred for the regeneration of cells and a continuous supply of factors is highly preferred. But as discussed above, in some cases burst release is encouraged for the release of drugs like DEX (Yao et al., 2019).

### 5.2 Sustained release

In cases where the prolonged release of drugs is required, this release kinetics is preferred. It helps in the delivery of the drug at a predetermined rate and this prolongs the period of release, thereby maintaining constant drug delivery (Li and Mooney, 2016). This kinetics is followed in cases where the lifespan of the drug in body fluids is very less and has a higher rate of elimination from the body. In the case of drug delivery for bone regeneration, this mode of kinetics is preferred as it aids for a long time until the neo bone formation.

### 5.3 Sequential release

The drug is delivered sequentially in the appropriate order of release. For example, in some cases, the drugs that help in preventing inflammation and that aid in osteogenic activity can be loaded. In such cases, the drug has to be released in such a way that the anti-inflammatory drug has to be released initially to prevent inflammation followed by the osteogenic drug as used by Zhenzhao Guo et al., in their research to guide bone regeneration (Guo et al., 2017).

### 5.4 Simultaneous release

In this approach, the drugs were released simultaneously. According to Lei Nie et al., VEGF and monocyte chemotactic protein-1 were employed to enhance angiogenesis for bone tissue engineering. These growth factors were encapsulated and delivered simultaneously in PLGA-mPEG microspheres (Nie et al., 2018).

### 5.5 Triggered release

It is designed in a way that the drug will be released from the scaffold only with the help of stimuli. The stimuli could be a triggering agent or the physicochemical changes than the normal physiological conditions (Barry and Rowski, 2002). It is not widely used in bone tissue engineering.

## 6 Types of dual drug delivery platforms for bone tissue engineering

**FIGURE 9:**
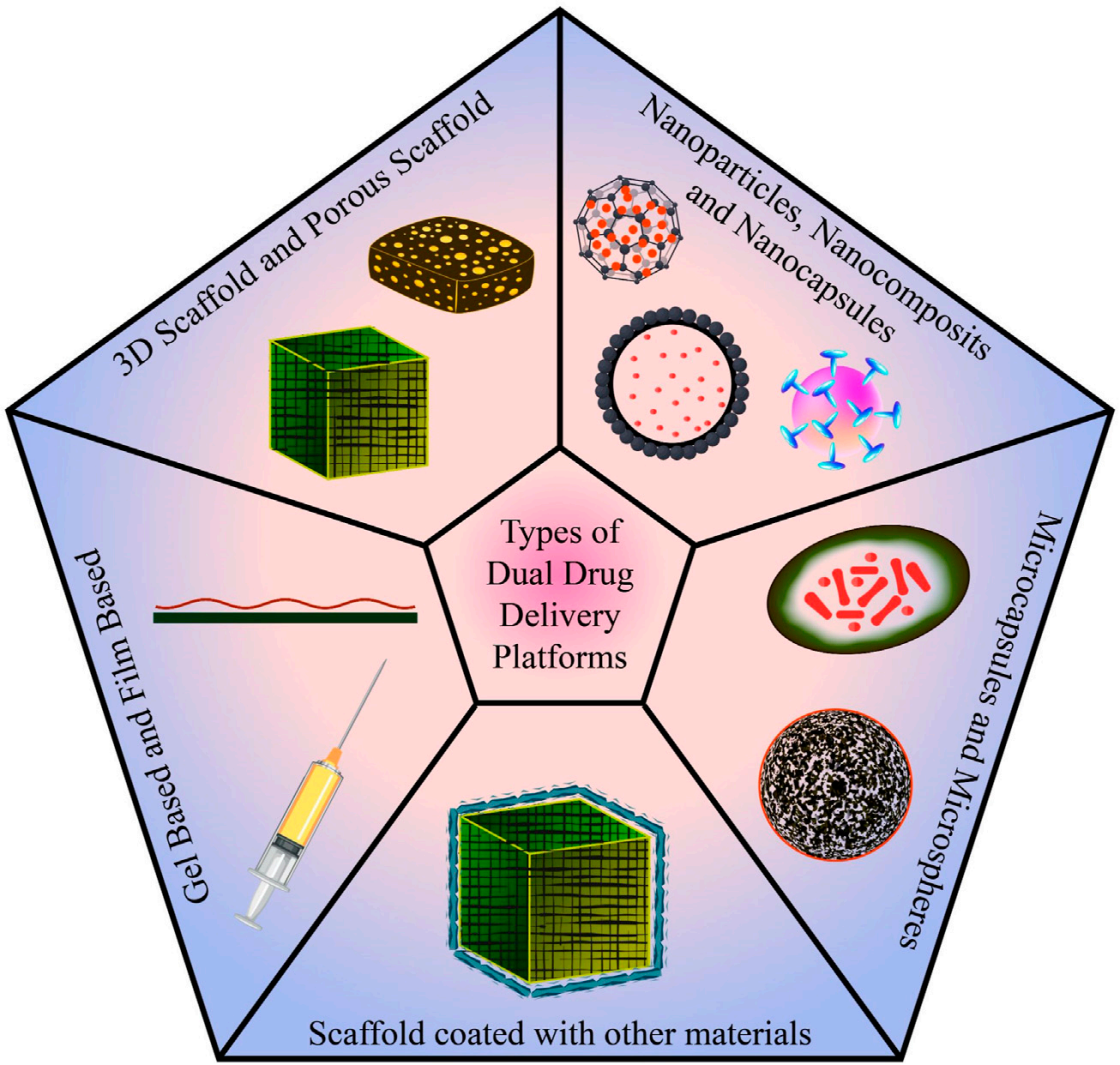
The five classes of dual drug delivery platforms.

The fabrication and design of the scaffold are critical to achieving an efficient and precise dual drug delivery platform. In the process, researchers worked on a wide range of bio-based materials such as biopolymers (alginates and chitosan), bio ceramics (calcium triphosphate and bio glass), gel and film-based systems (hydrogel and polyelectrolyte multilayer films), and other contemporary advancements like microsphere and microcapsule-based platforms, and nanoparticle-based scaffold (Figure 9).

In general, scaffold design incorporates 3D printing; however, in a few cases, 3D printing is intertwined with release and delivery, necessitating a separate characterization of 3D scaffolds. In tissue engineering, a 3D scaffold is used to structure the tissue, monitor cell characteristics, and deliver drugs. These are crafted with biodegradable, porous, and biocompatible materials that display superior attributes like enhanced mechanical strength and optimal niche with physical and morphogenetic stimuli for growth and development (Loh and Choong, 2013). The major platforms used for dual drug delivery in bone tissue engineering are classified and mentioned below.

### 6.1 3D Scaffold and porous scaffold

#### 6.1.1 PPF/Gelatin

For the study of the dual drug delivery of BMP-2 and VEGF, the researcher group of Albert K Shung used a scaffold made of porous poly (propylene fumarate) (PPF) containing gelatin microparticles (Patel et al., 2008). Two types of gelatin microparticles (acidic and basic) cross-linked with varying concentrations of glutaraldehyde were used for the release of VEGF and BMP-2, respectively. The technique of diffusional loading by dipping the lyophilized gelatin microparticles into the solution of VEGF and BMP-2 was employed for the fabrication of gelatin microparticles (Young et al., 2009). The PPF was synthesized in a two-step process where the structure was substantiated by Nuclear Magnetic Resonance (NMR) and also an average molecular weight was indicated along with a PPF number by gel permeation chromatography (Shung et al., 2002).

In another study carried out by Zarana S Patel et al., the scaffold was fabricated with a 1:1 proportion of N-vinyl pyrrolidone with 80% NaCl as a porogen to present a porous texture and increase efficiency (Patel et al., 2008). These porous PPF scaffolds and BMP-2 and VEGF-loaded gelatin microparticles were combined to develop a composite scaffold. The PPF received equal amounts of both acidic and basic gelatin microparticles. However, the amount of BMP, VEGF, and blank (PBS loaded) gelatin microparticles differed with the number of varied experimental groups. So, the regulating factor for varied dosages of BMP-2 and VEGF was the ratio of loaded to unloaded gelatin microparticles. The study was carried out in a rat calvaria model by Simon Young et al., where they concluded that the simultaneous release of BMP-2 and VEGF hadn’t increased the neo-bone formation when compared with the BMP-2 release alone (Young et al., 2009).

#### 6.1.2 NBBM

The dual drug administration of recombinant human bone morphogenetic protein-7 (rhBMP-7) and recombinant human vascular endothelial growth factor 165 (rhVEGF165) using a natural bovine bone mineral (NBBM) scaffold was studied by Yanming Liu and colleagues from Zhejiang University School of Medicine in China. The scaffold was designed with a mesh cage-like structure made of titanium and NBBM, that was further loaded with rhBMP-7 and rHVEGF_165_. For the experimental purpose, the six variants of loading in NBBM were used. These scaffolds were implanted in eight pigs to observe the *in-vivo* effects where fluorochromes were used to monitor bone formation in the early stages. The synergistic effect of the two bioactive factors, rhBMP-7 and rhVEGF165, in bone tissue regeneration and development was shown to be promising in this work (Liu Y et al., 2014).

#### 6.1.3 TCP/PLGA

**FIGURE 10:**
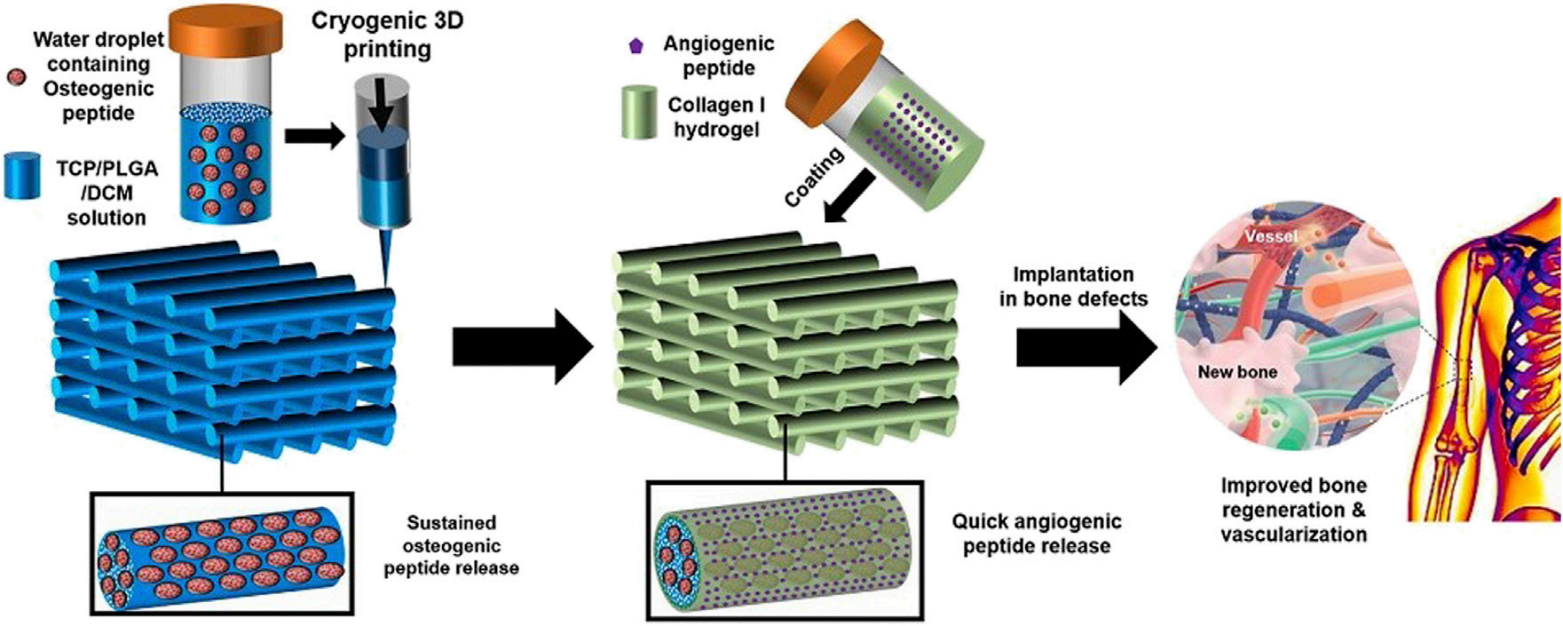
Diagrammatic representation of fabricating TCP/PLGA scaffolds via cryogenic 3D printing for delivering AP and OP with subsequent hydrogel coating. (Reproduced with permission from Ref (Wang et al., 2021) under Creative Commons Attribution License (CC BY)).

Primarily, a suspension was obtained by ultra-sonication of a mixture of PLGA and β-tricalcium phosphate (β-TCP) in DCM, then Osteogenic peptide (OP) was added to it, forming a white composite emulsion, which was employed as ink for 3D printing the scaffold. A cryogenic 3D printer was used to print the composite 3D scaffold with a pore diameter of 200 nm as depicted in Figure 10. Later, it was surface coated with an Angiogenic peptide (AP) infused collagen hydrogel. This platform resulted in the dual delivery of AP and OP sequentially. Upon *in-vitro* and *in-vivo* investigation of the scaffold, it was found to have significant improvement in bone regeneration and vascularization properties (Wang et al., 2021).

### 6.2 Gel-based and film based

#### 6.2.1 Heparin conjugated fibrin

For bone regeneration, a heparin conjugated fibrin (HCF) scaffold in gel form was designed by Hyun Sook Hong et al., to deliver BMP-2 and SP. SP is a neuropeptide essential for the growth of cells and their proliferation (Hong et al., 2009). On the other hand, BMP-2 is required for the osteogenic differentiation of mesenchymal stem cells (Mostafa et al., 2012).

The primary idea behind the synthesis of HCF is to covalently couple/conjugate bovine fibrinogen to heparin. It began with the dissolving of heparin in a buffer (2-morpholino ethane sulfonic acid), followed by the addition of N-hydroxy succinimide (NHS) and 1 ethyl 3 - (3 dimethyl aminopropyl) carbodiimide hydrochloride (EDC) to activate the heparin’s -COOH group. After an incubation (at 4°C for 12 h), it was stirred until a homogenous solution was achieved, followed by lyophilization of the homogenous solution and precipitation using anhydrous acetone. In the case of fibrinogen, a similar procedure was used, with fibrinogen being dissolved in phosphate buffer saline (PBS) and then treated with activated carboxylic groups of heparins. To make a white powder, it was precipitated and lyophilized. This was diluted in PBS again, and the excess heparin was removed by dialyzing through a porous membrane bag. After final lyophilization, the HCF was created by conjugating heparin conjugated fibrinogen in a 1:1 ratio with normal fibrinogen, which ensured superior mechanical strength and properties for HCF (Noh et al., 2015). This platform facilitated the rapid release of SP while reducing the release of BMP-2. Following *in-vivo* testing, the platform was found to have significantly improved bone regeneration properties (Noh et al., 2015).

#### 6.2.2 Polyelectrolyte multilayer films

For better control of the bone regeneration process, it is essential to facilitate the appropriate concentration of the correct growth factor at the correct time during the regeneration process. Polyelectrolyte multilayer (PEM) films were developed to facilitate the sequential release of rhBMP-2 for promoting osteogenesis and rhVEGF_165_ for promoting angiogenesis (Patel et al., 2008).

The construction of the film was preceded by the preparation of a polyelectrolyte solution. Poly (-amino ester) 2 (Poly2), poly (acrylic acid) (PAA), chondroitin sulfate, rhBMP-2, and rhVEGF_165_ were the different polyelectrolytes or dipping solutions. All these dipping solutions were prepared using sodium acetate buffer with varying concentrations of the materials (Lynn and Langer, 2000; Shah et al., 2011).

A macroporous polycaprolactone/β-TCP waffle cylinder scaffold was sliced into two halves with a razor blade for film fabrication, and a plasma etched with room air was quickly immersed in Poly2. Primarily, a tetra layer film was constructed for the first growth factor rhBMP-2, starting with the fabrication of rhBMP-2 nanolayered films, following the dipping protocols, where it was washed with Poly2, PAA, rhBMP-2 and again with PAA solution. This was repeated until a tetralayer was achieved. The second growth factor, rhVEGF165, was synthesized using similar techniques but with rhBMP-2 dipping solution replaced with rhVEGF165 and PAA replaced with chondroitin sulfate. In addition, single growth factor films were created as controls. If not released or implanted, these films are kept at 4°C (Ai, Jones and Lvov, 2003; Tang et al., 2006; Shah et al., 2011).

The release studies showed that VEGF had a burst release in the first 8 days thereby upregulating proliferation, whereas BMP-2 had a sustained release for 2 weeks aiding the differentiation of cells. *In-vivo* studies showed that the bone regenerated had a higher thickness and better vascularization, hence reducing the chances of osteoporotic fracture. This platform is also found to be suitable for dual drug delivery in a dose-dependent manner, and hence holds the potential for precise dosage delivery of multiple drugs at a time. (Shah et al., 2011).

### 6.3 Scaffold coated with other materials

#### 6.3.1 Mineral coated porous β-TCP

Calcium phosphate ceramics are very popular for their usage as bone substitutes due to their property of being bioactive and osteoconductive, Beta tricalcium phosphate is one of the most famous biomaterial for this purpose (Sohier et al., 2010). The β-TCP scaffold was modified by coating it with a mineral layer without hindering the dimensions and porosity. This scaffold was loaded with rhVEGF and a modular peptide variant of BMP-2 with a sequence that increases its affinity for mineral binding (Saito et al., 2004, 2006; Lee, Wagoner Johnson and Murphy, 2010), to promote angiogenesis and osteogenesis respectively (Suárez-González et al., 2014).

The scaffold was built using a pristine β -TCP ceramic suspension and indirect solid free-form fabrication methods. In other words, a scaffold with a 6 × 4 mm dimension and a 753 nm pore size was structured utilizing image-based design and 3D printing. First, a mixture was prepared with 40% by volume β-TCP powder along with the required deflocculants and acrylate binder. This mixture or slurry was cast into a design mold (generated via Model Maker II) and was taken out, following the ceramic curing. The burnout scaffolds were air sintered for 5 hours at 1,100°C. Following incubation, the scaffolds were dipped in SBF solution containing twice the calcium and phosphate concentrations of human plasma to stimulate growth (Suárez-González et al., 2010). Subsequently, a batch of reagents were added to it in this sequential order: dH_2_O, NaCl, KCl, MgSO_4_, MgCl_2_, NaHCO_3_, HEPES, CaCl_2_, KH_2_PO_4,_ and the pH was optimized to 6.8. Following this, the scaffold was observed under SEM. Finally, the mineral-coated scaffold was sputter-coated with gold nanoparticles after mounting on aluminium stubs, and was imaged under Scanning Electron Microscopy (SEM). The growth factors were loaded onto the scaffold by dipping it sequentially in the rhVEGF and BMP-2 solutions. The release profile substantiates the release as a sustained release for up to 60 days for both the growth factors. Also, the concentration of rhVEGF was found to be higher than the concentration of BMP-2, demonstrating the fact that BMP-2 has a higher affinity toward the mineral in the scaffold (Suárez-González et al., 2014). The *in-vivo* studies in sheep showed enhanced blood vessel growth by the influence of rhVEGF in 2 weeks, and there was an amplified infiltration of tissue into the scaffold due to the influence of mBMP-2 after 4 weeks (Suárez-González et al., 2014).

### 6.4 Microcapsules and microspheres

#### 6.4.1 PLGA/PPF/Gelatin

**FIGURE 11:**
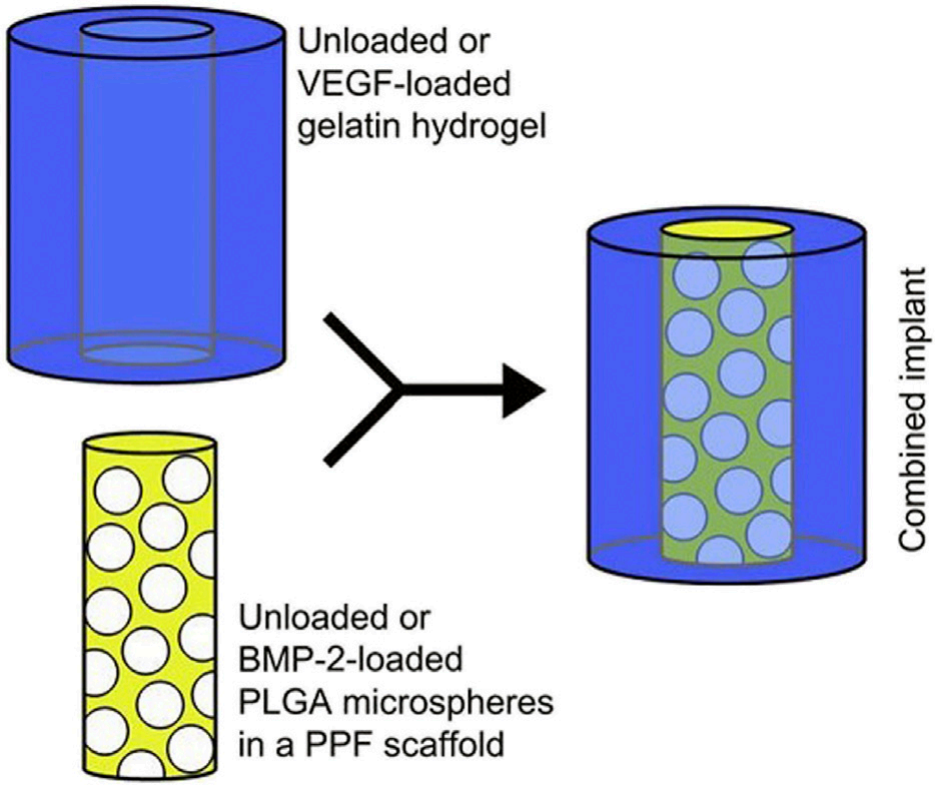
A composite scaffold for the sequential delivery of VEGF and BMP-2 is illustrated. (Reproduced with permission from Ref (Kempen et al., 2009). Copyright ^©^ 2009 Published by Elsevier Ltd.).

A dual drug delivery platform of PPF containing microspheres of PLGA which was circumscribed by hydrogel was designed for the sequential delivery of VEGF and BMP-2 as represented in Figure 11 (Kempen et al., 2009).

BMP-2 was loaded into PLGA microspheres using a water-in-oil-in-water double-emulsion-solvent-extraction approach to initiate the composite scaffold fabrication (Oldham et al., 2000; Kempen et al., 2008). These microspheres were placed in a PPF cylinder, which was fabricated through photo crosslinking of PPF in a two-step process (Wang, Lu and Yaszemski, 2006). The outer hydrogel was procured from gelatin and was fabricated separately in an optimum and sterile condition, and the VEGF was impregnated into this hydrogel to develop it into a dual delivery composite scaffold (Kempen et al., 2008).

*In-vivo* investigations in the rat model revealed an initial burst release of VEGF within the first 3 days due to rapid degradation of the platform carrying it (i.e., hydrogel) and a 56-days sustained release of BMP-2. Although the release of VEGF alone had little effect on bone regeneration, it did increase the action of BMP-2 in the ectopic bone regeneration process. The findings were inconclusive, necessitating more detailed research (Kempen et al., 2009).

#### 6.4.2 PLGA/P4VN/Alginic acid

In this dual drug delivery platform, the scaffold was constructed by the incorporation of microspheres into PLGA. The microspheres were designed by the combined effect of crosslinking and complexation of polyelectrolyte in calcium chloride (CaCl_2_) solution (Darvari and Hasirci, 1996; Buket Basmanav, Kose and Hasirci, 2008). Poly(4-vinyl pyridine) (P4VN) and Alginate–Bovine serum albumin (BSA) solutions of similar concentration were blended in a 3:1 ratio and the same was dropped into CaCl_2_ solution, generating microspheres, which are washed and freeze dried prior to use. After procuring the microspheres (P4VN–alginic acid), they were placed in the mold, and PLGA solution was poured into it, resulting in the entrapment of the microspheres (Ulubayram et al., 2001; Perets et al., 2003). The porosity of the scaffold was ensured by a mercury porosimeter.

When the platform was loaded with single and double microspheres, a variation in pore size was observed. Rat bone marrow-derived stem cells (BMSC) were isolated, and it was discovered that the presence of both microspheres increased cell proliferation to its highest level. It was found that the addition of BMP-2 and BMP-7 to the drug delivery platform resulted in a decrease in cell proliferation and an increase in differentiation. Because of this, simultaneous release of BMP-2 and BMP-7 is more effective than a single injection of BMP-2 or BMP-7 at promoting cell differentiation and enabling bone healing (Buket Basmanav, Kose and Hasirci, 2008).

### 6.5 Nanoparticle-based, nanocomposite and nanocapsules

#### 6.5.1 BSA/PCE

**FIGURE 12:**
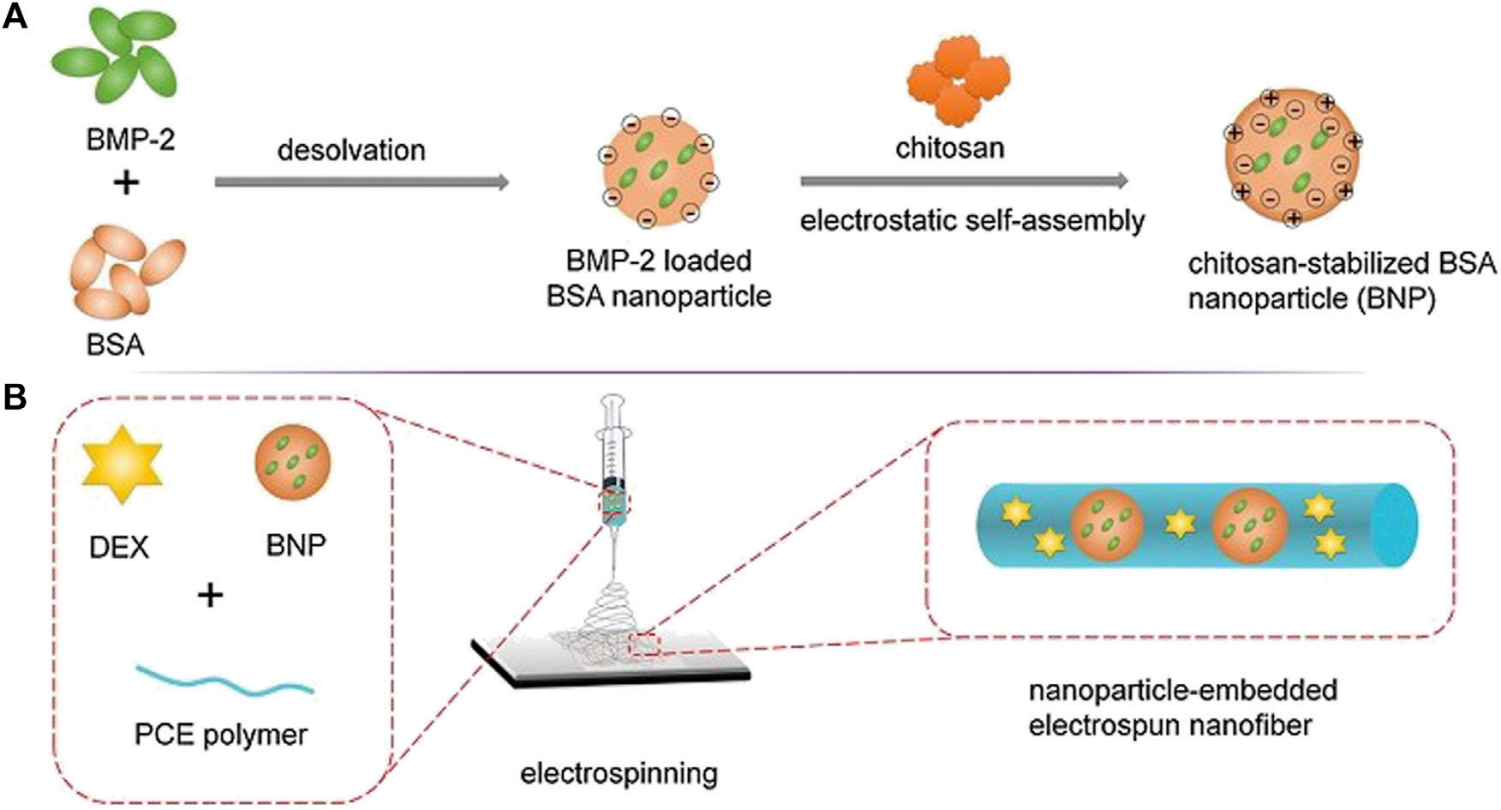
Illustration depicting the **(A)** Synthesis of BMP-2 loaded BSA nanoparticles **(B)** Nanoparticle embedded electro spun nanofiber (Reproduced with permission from Ref (Li et al., 2015). Copyright ^©^ 2014 Elsevier Ltd.).

This platform was designed as in Figure 12, in an attempt to promote biomimetic bone tissue growth using BMP-2 and DEX. The scaffold was composed of electrospun nanofiber with nanoparticles embedded in it. BMP-2 is one of the most widely employed bioactive molecules for bone regeneration, primarily to induce mesenchymal stem cell differentiation into the osteogenic lineage. DEX, on the other hand, has similar potential and function to BMP-2. However, DEX should not be used indefinitely due to its harmful side effects (Oshina et al., 2007). But, the problem with BMP-2 is that it has a short half-life and hence, it is not always successful for bone regeneration therapy (Takahashi, Yamamoto and Tabata, 2005). Owing to the hindsight of the BMP-2 and DEX, this platform was developed to overcome them (Li et al., 2015).

The polymerization of PEG using stannous octoate (Sn (Oct)2) as a catalyst resulted in the synthesis of Poly (-caprolactone) -poly (ethylene glycol) (PCE). The BSA nanoparticles were then synthesized and BMP-2 was loaded to create BMP-2 loaded chitosan stabilized BSA nanoparticles. Following that, electrospinning technique is used to prepare scaffolds. PCE, NPs/PCE, BNPs/PCE, DEX/PCE, and BNPs/DEX/PCE were the five scaffolds constructed (Li et al., 2015).

The nanoparticles containing scaffolds were prepared by using an ink made of ultrasonically redispersed nanoparticles mixed in Dimethylformamide (DMF), PCE, and DCM, which was electrospun. Other scaffolds were immobilized with DEX and BNPs/DEX/PCE, both of which were fabricated by electrospinning a mixture of DEX, DCM, PCE, and DMF, except the latter containing nanoparticles (Li et al., 2015).

The studies revealed that a release pattern in which each item is released one at a time with an initial burst release of DEX was observed every 8 days, followed by a sustained release of BMP-2 for 35 days. The *in-vivo* studies on a rat for a calvaria defect showed efficient bone regeneration, in which the early stage of bone regeneration was credited to the action of DEX, and the later stages of bone formation are credited to BMP-2 (Li et al., 2015).

#### 6.5.2 Chitosan/PLGA/PHBV

In the scaffold preparation, the first step was the preparation of BMP-2 and BMP-7 coated nanoparticles. In dichloromethane containing PLGA and Poly(3-hydroxybutyrate-co-3-hydroxyvalerate) (PHBV), BMP or BSA aqueous solutions were emulsified and then transferred into PVA. This technique involves double emulsion solvent evaporation (Yilgor et al., 2010a). Then the nanoparticles were procured after washing, and centrifugation, followed by lyophilization (Yilgor et al., 2009). The next step was to design the chitosan-based 3D fibrous scaffold which had a mesh-like texture and was designed by employing wet spinning of the mixture of chitosan/PEO and chitosan. The mixing of chitosan and PEO was done to test the mechanical durability of the construct (Yilgor et al., 2009).

The sequential release pattern of BMP-2 and BMP-7 was most effective when their nano capsules were linked to the 3D fibrous scaffold, based on *in-vitro* investigations. These two growth factors were investigated for single, sequential, and simultaneous delivery, and it was observed that sequential delivery of BMP-2 and BMP-7 increased ALP activity while simultaneous delivery had no impact on cell number or activity, but the single delivery alone inhibited cell proliferation. As a result, the platform described above could be one of the most effective techniques for dual drug administration in bone tissue engineering, thanks to its resemblance to the natural process of bone regeneration (Yilgor et al., 2009).

#### 6.5.3 SF/PCL/PVA

**FIGURE 13:**
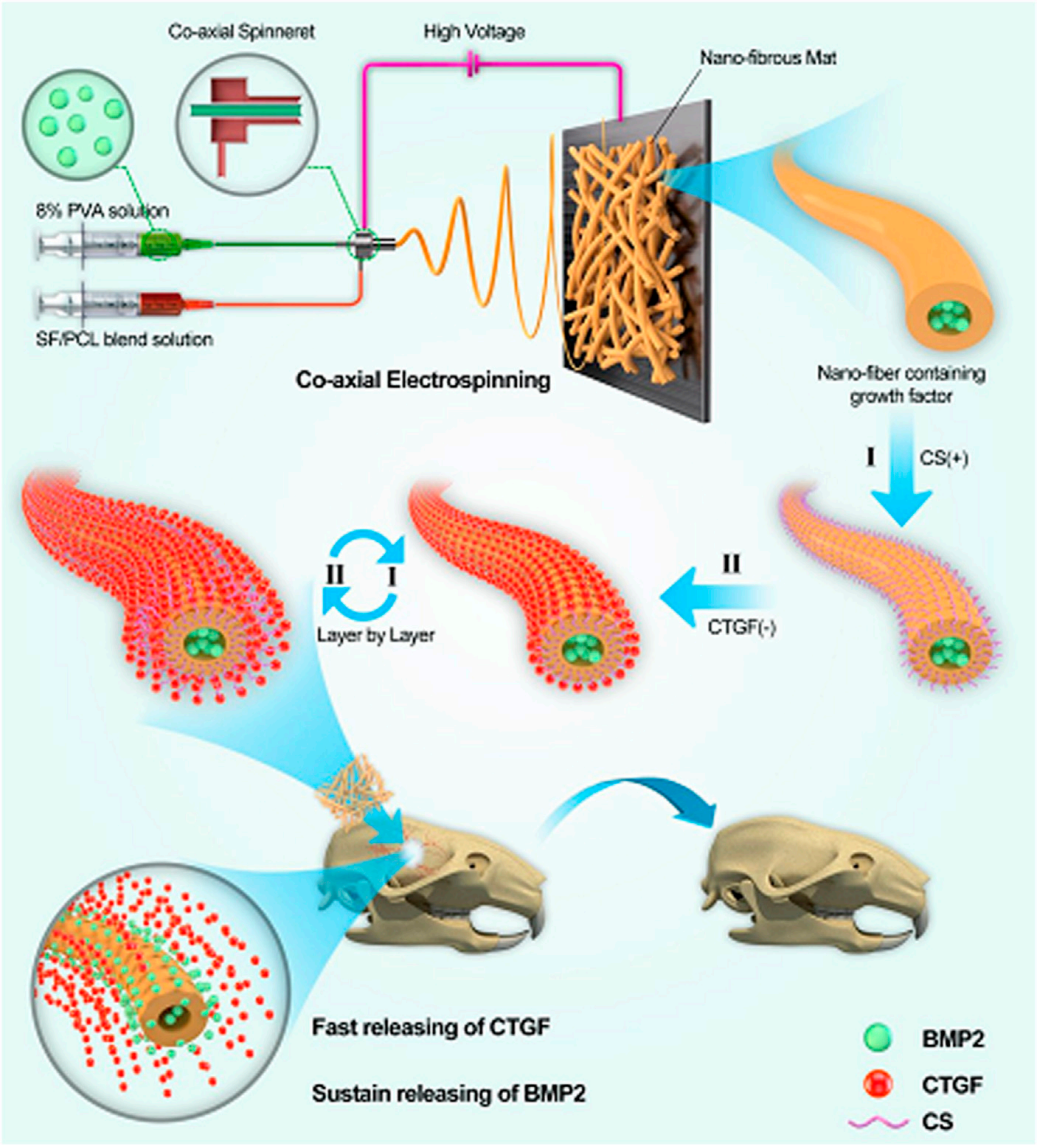
Schematic illustration showing the incorporation of BMP-2 and CTGF in the core-shell nanofibers of SF/PCL/PVA mats used for bone tissue engineering. (Reproduced with permission from Ref. (Cheng et al., 2019). Copyright 2019, American Chemical Society).

As shown in Figure 13, a nanofibrous core-shell mat was created by electrospinning of SF/PCL/PVA for the time-controlled delivery of BMP-2 in the core and the swift release of connective tissue growth factor (CTGF) in the shell of nanofibers. The mat has been employed for bone tissue engineering applications, and *in-vitro* and *in-vivo* results revealed that these nanofibers increased bone regeneration by 43% when compared to BMP-2 administration alone. The slow release of BMP-2 thus ensured that it remains during the entire period of the bone healing process. The time - dependent release accompanied by the rapid delivery of the two growth factors thus facilitated to be as a promising strategy for enhanced bone regeneration (Cheng et al., 2019).

Although this strategy is promising, further research on other growth factors that ensures bone healing can be carried out and more recent technologies could be incorporated to fabricate the nanocomposite fibers.

Table 2 is a summary of numerous platforms for drug delivery in bone tissue engineering applications constructed with diverse biomaterials.

**TABLE 2:** Summary of various biomaterial based platforms used for dual drug delivery.

S No	Platform	Material	Drugs	References
1	3D Ceramics	Hydroxyapatite	BMP-2 and DGEA	Chen et al. (2020)
2	Nanofibers	SF/PCL/PVA	BMP-2 and CTGF	Cheng et al. (2019)
3	Granules	Biomimetic calcium phosphate bone substitute	BSA and BMP-2	Liu T et al. (2014)
4	Gel based	Chitosan/PVA hydrogel and poly(L-glutamic acid) micelles	Aspirin, doxorubicin	Wei et al. (2009)
5	Nanocomposites	SF/PLGA nanofibers	RhBMP-2, DEX	Guo et al. (2017)
6	Nanocomposites	Mesoporous silicate nanoparticles incorporated-3D nanofibrous gelatin	Deferoxamine and BMP-2	Yao et al. (2018)
7	3D Scaffold	PLLA	Parthenolide, naringin	Guo et al. (2017)
8	Porous scaffold	Agarose/nHCA	Ibuprofen, zoledronic acid	Paris et al. (2015)
9	Film based	Aldronate sodium trihydrate, PGLA, PAA, Poly2	BMP-2 and PDGF-BB	Shah P et al. (2014)
10	Nano capsules	PLGA and PHBV	BMP-2 and BMP-7	Yilgor et al. (2009)
11	Porous Scaffold	PHBV and Chitosan	PDGF-BB and BMP-6	Demirtaş et al. (2016)
12	Microparticles	Chitosan and PLGA	Vancomycin and rhBMP-2	Song and Xiao, (2021)
13	Nanoparticles in 3D scaffold	PLGA and PHBV/PCL	BMP-2 and BMP-7	Yilgor et al. (2010b)
14	Microspheres	PLGA	VEGF and BMP-2	Hernández et al. (2012)
15	Microcapsules	PLGA and Alginate	DEX and BMP-2	Choi et al. (2010)
16	Nanomicelle composites	Mesoporous bioactive glass/polypeptide graft copolymer	Gentamicin, naproxen	(Xia et al., 2008
				Wu and Chang, (2012)
17	3D Scaffold	PPF and Gelatin	VEGF and BMP-2	Young et al. (2009)
18	Porous Scaffold	NBBM	rhBMP-7, rHVEGF_165_	Liu Y et al. (2014)
19	3D Scaffold	TCP and PLGA	AP and OP	Wang et al. (2021)
20	Gel based	HCF	BMP-2, SP	Noh et al. (2015)
21	Film based	PEM films	rhBMP-7, rHVEGF_165_	Shah et al. (2011)
22	Mineral coated scaffold	β-TCP	rhVEGF and BMP-2	Suárez-González et al. (2014)
23	Microspheres	PLGA/PPF/Gelatin	VEGF and BMP-2	Kempen et al. (2009)
24	Microspheres in 3D scaffold	PLGA P4VN + Alginic acid	BMP-2 and BMP-7	Buket Basmanav, Kose and Hasirci, (2008)
25	Nanocomposite	BSA/PCE	BMP-2 and DEX	Li et al. (2015)
26	Nanoparticles	Chitosan and PLGA + PHBV	BMP-2 and BMP-7	Yilgor et al. (2009)

## 7 Conclusion and future prospects

It is a well-known fact that natural processes are intricate and involve a cascade of reactions linked with each other. Dual drug delivery is an attempt to mimic the natural process of bone regeneration to enhance the process. Conventional techniques for treating bone-related damages are very popular but they manifest certain disadvantages like adverse side effects and low selectivity of the treatment. Some treatment requires repeated surgeries and most patients tend to avoid such invasive treatment. Tissue-engineered scaffolds for bone tissue engineering are evolving day by day as it provides physical support and promotes various biological processes required for bone regeneration. Dual drug delivery is a step forward in this direction, with a lot of promise for treating large and complex bone injuries by ensuring the sequential, precise, and controlled release of growth factors, antibiotics, and other bioactive molecules to the target site of cells involved in the bone regeneration process.

Biological systems are indeed very complex, and many researchers in an attempt to develop a dual drug delivery platform reported the complex nature and the developed platform not to be very effective as expected theoretically. Many researchers got promising results in *in-vitro* studies but *in-vivo* studies were not very encouraging. Hence, there is a lot to discover and a lot to establish in this domain. This breakthrough in bone tissue engineering has good prospects and could be used for therapeutic purposes soon. Not just dual drug delivery, the scientific community must contribute towards the development of multidrug delivery platforms, aiming to deliver more than two drugs precisely and sequentially, and maybe developing a platform that is capable of delivering drugs on demand.

## Abbreviations

ECM, Extra cellular matrix; PDGF, platelet-derived growth factor; FGF, fibroblast growth factors; TGF-β, transforming growth factor beta; IGF, insulin-like growth factor; BMP, bone morphogenetic proteins; FDA, Food and Drug Administration; SIBLINGS, small integrin-binding ligands; OCN, osteocalcin; MGP, matrix Gla protein; SLRPs, small leucine-rich proteoglycans; TSP, thrombospondins; BSP, bone sialoprotein; OPN, osteopontin; DMP-1, dentin matrix protein-1; DSPP, dentin sialophosphoprotein; MEPE, matrix extracellular phosphoglycoprotein; HA, hydroxyapatite; kDa, KiloDalton; WHO, World Health Organization; HGF, hepatocyte growth factor; PLGA, poly (lactic-co-glycolic acid); IL, interleukin; TNF, tumor necrosis factor; DEX, Dexamethasone; CXC, chemokine; SP, Substance P; PEG, Polyethylene glycol; PPF, poly (propylene fumarate); NMR, Nuclear Magnetic Resonance; PBS, Phosphate Buffer saline; OP, Osteogenic peptide; NBBM, Natural Bovine bone mineral; TCP, tricalcium phosphate; AP, Angiogenic peptide; HCF, heparin conjugated fibrin; NHS, N-hydroxy succinimide; EDC, 1-ethyl 3 -(3 dimethylaminopropyl) carbodiimide hydrochloride; PAA, poly (acrylic acid); Poly2, Poly (-amino ester) 2; CaCl_2_, Calcium chloride; BSA, Bovine serum albumin; BMSC, bone marrow derived stem cells; MSC, Mesenchymal stem cells; PHBV, poly(3-hydroxybutyrate-co-3-hydroxyvalerate); PCE, poly (-caprolactone) - poly (ethylene glycol); DMF, dimethylformamide; P4VN, poly(4-vinyl pyridine); CTGF, connective tissue growth factor; PEM, polyelectrolyte multilayer; SF, silk fibroin.
